# Supplementary figures and images for: Biogeography and Genetic Diversity of Terrestrial Mites in the Ross Sea Region, Antarctica
Source: Genes (Basel). 2023 Feb 28;14(3):606. doi: 10.3390/genes14030606 (PMC10048765; doi:10.3390/genes14030606)

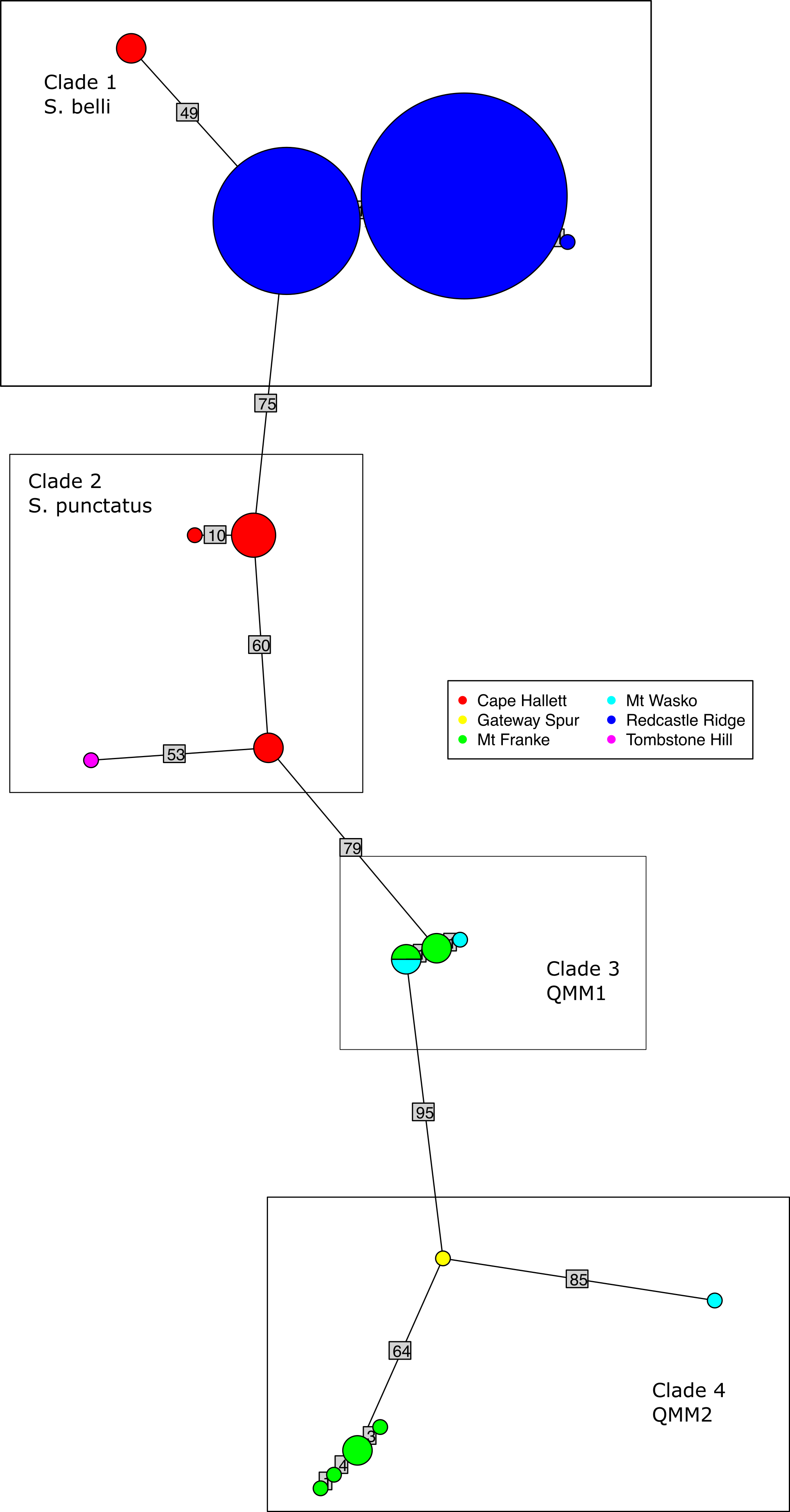

Supplement: Supplementary file 1 [file genes-14-00606-s001.zip › Supplementary figure 1_Stereotydeus_network_modified.png]

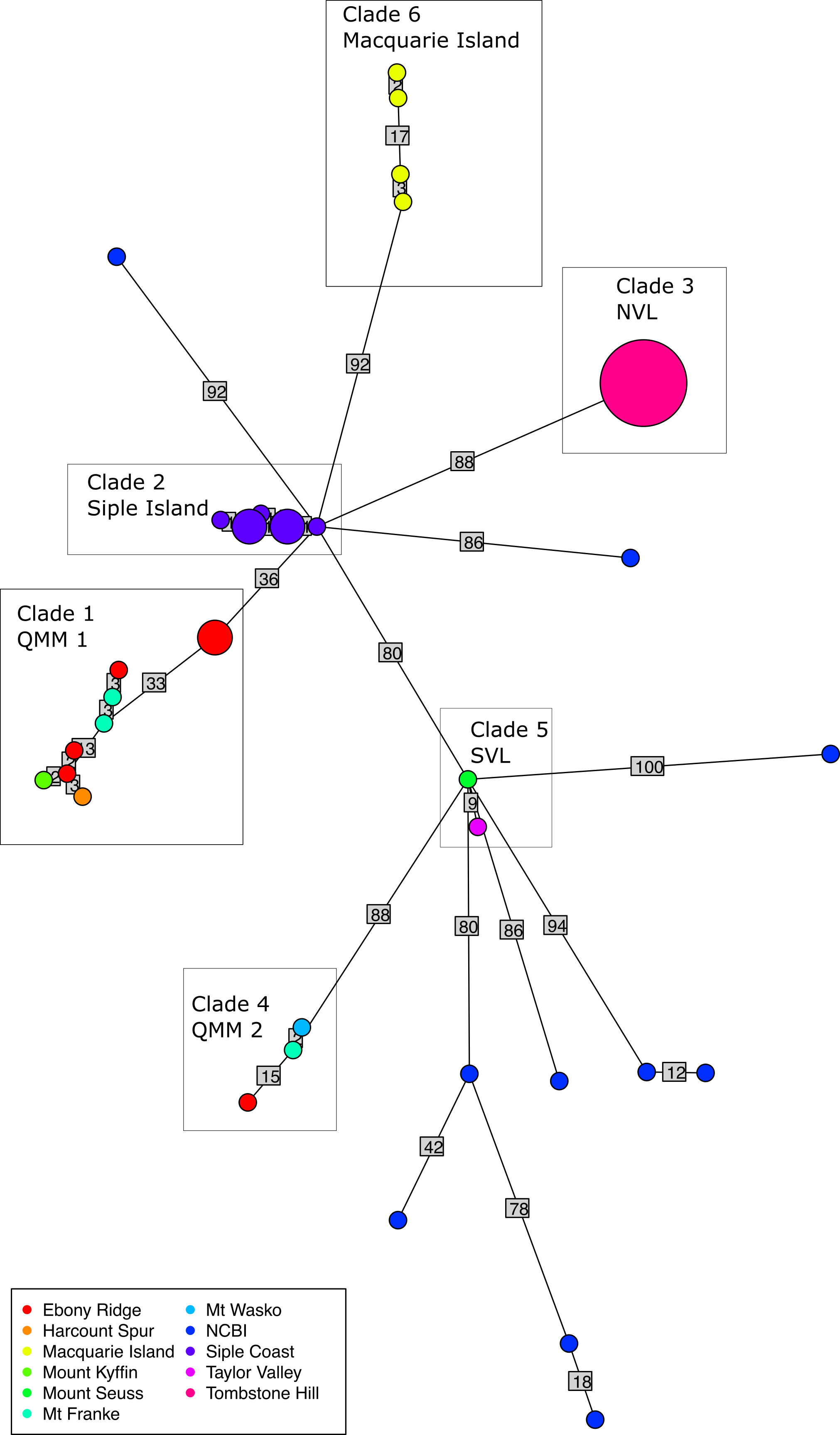

Supplement: Supplementary file 1 [file genes-14-00606-s001.zip › Supplementary figure 2_Nanorchestes_network_modified.png]

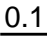

- Sub-Antarctic

Supplement: Supplementary file 1 [file genes-14-00606-s001.zip › Supplementary figure 3_full tree.pdf]
